# Enhancing Sealant Retention: The Role of Erbium-Doped Yttrium Aluminum Garnet (Er:YAG) Laser Preconditioning Under Saliva-Contaminated Conditions

**DOI:** 10.7759/cureus.96128

**Published:** 2025-11-05

**Authors:** Varalakshmi C, Lokesh B Kanchan, Rachana P Hiremath, Anusha Timmapur, Vidyananda Biradar, Akshay V Anand

**Affiliations:** 1 Conservative Dentistry and Endodontics, Hyderabad Karnataka Education Society’s (HKES) S. Nijalingappa Institute of Dental Sciences and Research, Kalaburagi, IND; 2 Prosthodontics, Crown and Bridge, Employees’ State Insurance Corporation (ESIC) Dental College, Kalaburagi, IND; 3 Paediatric and Preventive Dentistry, Employees’ State Insurance Corporation (ESIC) Dental College, Kalaburagi, IND; 4 Conservative Dentistry and Endodontics, Al Badar Rural Dental College and Hospital, Kalaburagi, IND; 5 Conservative Dentistry and Endodontics, Vokkaligara Sangha Dental College and Hospital, Bengaluru, IND

**Keywords:** clinically challenging scenarios, er:yag laser, fissure sealant, saliva contamination, sealant retention

## Abstract

Introduction

Laser-assisted enamel conditioning combined with acid etching has shown potential in enhancing micromechanical retention. However, most prior studies were conducted under ideal dry conditions, overlooking the impact of saliva contamination, a common clinical challenge during sealant placement.

Objective

This study aimed to evaluate the effect of erbium-doped yttrium aluminium garnet (Er:YAG) laser pre-conditioning and saliva contamination on the shear bond strength (SBS) of a hydrophilic fissure sealant, *UltraSeal XT® hydro™.*

Methods

Forty extracted human mandibular molars were randomly divided into four groups (n = 10). Group 1 received acid etching only, Group 2 underwent acid etching followed by saliva contamination, Group 3 was treated with Er:YAG laser followed by acid etching, and Group 4 received Er:YAG laser, acid etching, and subsequent saliva contamination.

The sealant was applied following treatment, after which all samples underwent thermocycling. SBS was tested using a universal testing machine, and the results were analyzed using descriptive statistics, one-way ANOVA, and Tukey’s post hoc test.

Results

Group 3 demonstrated the highest SBS (mean: 22.3 MPa), significantly outperforming the other groups (p < 0.001). Group 2 exhibited the lowest SBS (mean: 19.6 MPa). Group 4 showed improved SBS compared to Group 2, suggesting partial mitigation of moisture effects by laser pre-conditioning.

Conclusion

Within the limitations of this in vitro study, Er:YAG laser pre-conditioning significantly enhances the SBS of hydrophilic sealants, particularly under saliva-contaminated conditions, offering a promising strategy for improving sealant retention in clinically challenging scenarios.

## Introduction

Dental caries remains one of the most prevalent chronic conditions worldwide, with occlusal surfaces of molars being particularly susceptible due to their deep pits and fissures that favor bacterial accumulation and plaque retention [1]. The placement of pit and fissure sealants has been widely recognized as an effective preventive strategy to protect these susceptible sites. However, the long-term success of sealants depends largely on the quality of adhesion between the resin and the enamel surface. Any compromise in this bond can lead to marginal leakage, sealant loss, and eventual caries development beneath the restoration. Clinical studies have reported sealant retention rates of approximately 70-90% after one year, with failure rates increasing over time due to factors such as inadequate adhesion or moisture contamination [2].

Traditionally, acid etching with 35-37% phosphoric acid has been considered the gold standard for enamel surface preparation prior to sealant application. The process creates microporosities that enhance micromechanical retention. However, despite its effectiveness, the bonding process is highly technique-sensitive. In clinical settings, particularly among pediatric or uncooperative patients, achieving and maintaining a dry field during sealant placement can be difficult. Moisture contamination, especially from saliva, can significantly impair the etching pattern, weaken resin infiltration, and ultimately reduce bond strength and sealant longevity [3,4].

To address these limitations, hydrophilic sealants such as UltraSeal XT® hydro™ have been introduced. These materials are formulated with moisture-tolerant chemistry, theoretically enabling bonding even in slightly moist conditions. Nevertheless, evidence regarding their actual performance under moisture contamination remains inconsistent [5]. Recent research indicates that while these sealants may exhibit improved wetting ability, bond strength still decreases in the presence of saliva, suggesting that further improvement in enamel pre-treatment protocols may be necessary [6].

In parallel, advancements in laser dentistry, particularly with the erbium-doped yttrium aluminium garnet (Er:YAG) laser, have provided an alternative approach for enamel surface modification. The Er:YAG laser emits at a wavelength of 2,940 nm, corresponding closely to the absorption peak of water and hydroxyapatite, allowing for efficient enamel ablation with minimal thermal damage [7]. Laser irradiation produces micro-roughened, prism-free enamel surfaces that may improve mechanical interlocking with resin materials and enhance bond durability [8,9]. Moreover, combining Er:YAG laser pre-conditioning with conventional acid etching has been reported to increase surface energy, improve wettability, and enhance resin tag penetration [10]. However, most studies investigating laser-assisted conditioning have been conducted under ideal, moisture-free conditions, leaving its performance in clinically realistic wet environments largely unexplored [11,12].

Therefore, this study aimed to evaluate the effect of Er:YAG laser pre-conditioning on the shear bond strength (SBS) of a hydrophilic pit and fissure sealant (UltraSeal XT® hydro™) under both dry and saliva-contaminated conditions. By simulating clinical challenges of moisture exposure, this research sought to determine whether laser pre-conditioning could mitigate the negative effects of contamination and thereby improve the long-term retention of hydrophilic sealants.

## Materials and methods

This in vitro study utilized forty sound, caries-free human mandibular molars extracted for periodontal, orthodontic, or surgical reasons and subsequently stored in 10% formalin solution until use. Prior to the experimental procedures, all samples were thoroughly rinsed with distilled water to remove any residual formalin. Teeth with fractures, restorations, caries, sealants, or developmental defects were excluded. Each tooth was sectioned at the cementoenamel junction using a slow-speed diamond disc, and crown portions were embedded in acrylic resin blocks with the buccal surface oriented upward.

Group allocation

The specimens were randomly divided into four groups (n = 10 per group). Group 1 received phosphoric acid etching followed by the application of UltraSeal XT® hydro™. Group 2 underwent phosphoric acid etching followed by artificial saliva contamination using Xerostat™ and subsequent UltraSeal XT® hydro™ application. Group 3 received Er:YAG laser pre-conditioning followed by phosphoric acid etching and UltraSeal XT® hydro™ application. Group 4 underwent Er:YAG laser pre-conditioning, phosphoric acid etching, and artificial saliva contamination using Xerostat™, followed by UltraSeal XT® hydro™ application.

Sample size determination

The sample size was estimated based on previously published in vitro studies evaluating the SBS of enamel conditioned with Er:YAG laser and conventional acid etching methods [7,10,12]. A power analysis was conducted using G*Power version 3.1 (Heinrich-Heine-University, Düsseldorf, Germany). Considering an effect size (f) of 0.60, an alpha error probability of 0.05, and a desired power (1-β) of 0.80, the minimum required sample size was calculated as eight specimens per group. To enhance data reliability and account for potential specimen loss during preparation or testing, ten samples were included per group, yielding a total of forty specimens.

Artificial saliva contamination protocol

Xerostat™ (ICPA Health Products Ltd., India) was used as the artificial saliva, containing sodium carboxymethylcellulose, sorbitol, potassium chloride, sodium chloride, magnesium chloride, calcium chloride, and potassium dihydrogen phosphate. The saliva was applied using a micropipette over the etched enamel surface for 10 seconds and gently air-dried for 5 seconds.

Laser conditioning

**Figure 1:**
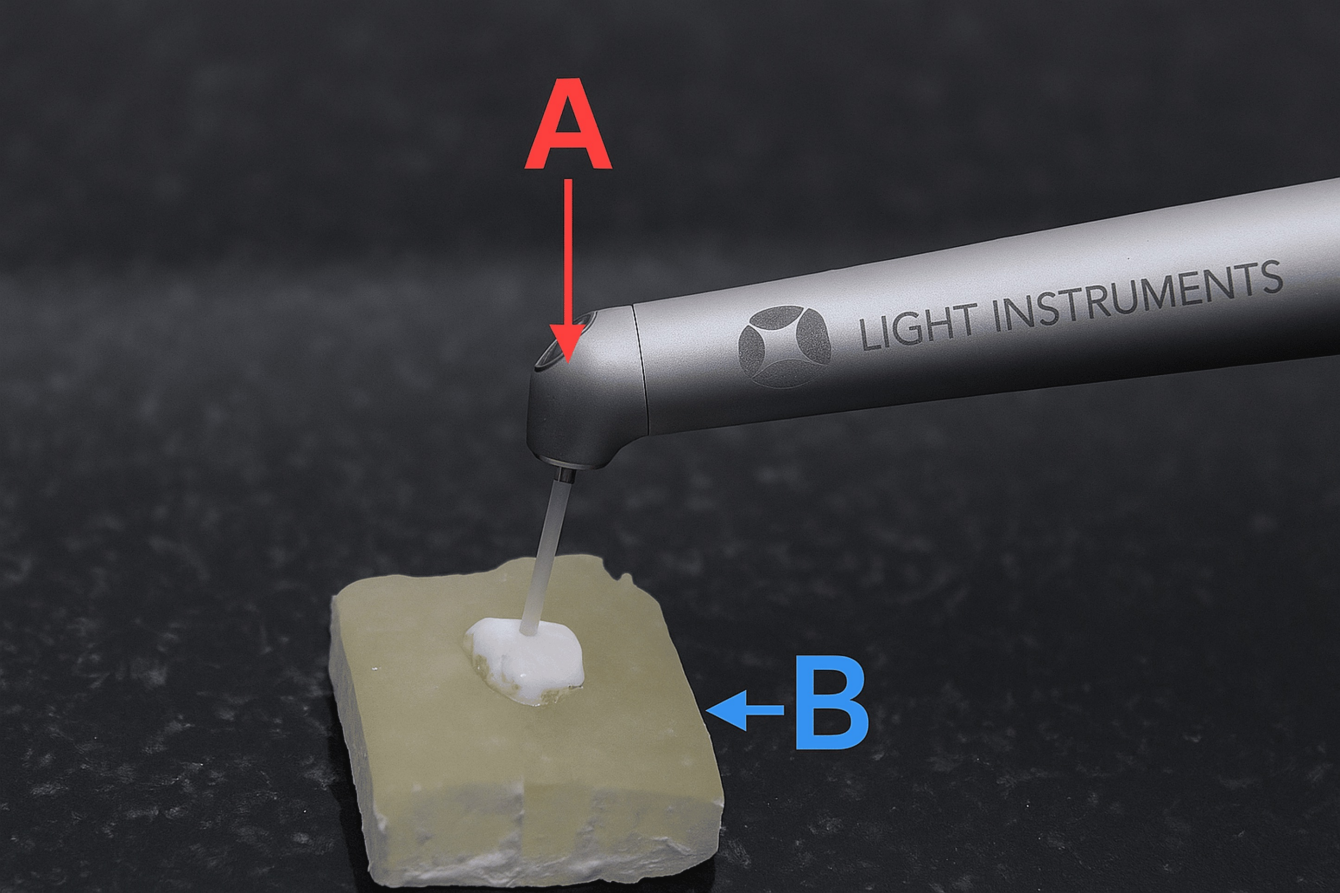
Laser irradiation of the enamel surface using the LiteTouch® Er:YAG laser system. (A) The red arrow indicates the Er:YAG laser handpiece directing the laser beam toward the enamel surface. (B) The blue arrow indicates the tooth specimen (buccal enamel surface) embedded in an acrylic resin block. Er:YAG: Erbium-Doped Yttrium Aluminum Garnet.

Laser irradiation was performed using the LiteTouch® Er:YAG laser system (2940 nm, spot size 0.9 mm; 120 mJ, 10 Hz, 1.2 W) with air-water cooling. The laser beam was directed perpendicular to the buccal surface in non-contact mode from a 1 mm distance for 15 seconds (Figure 1).

Bond strength testing

**Figure 2:**
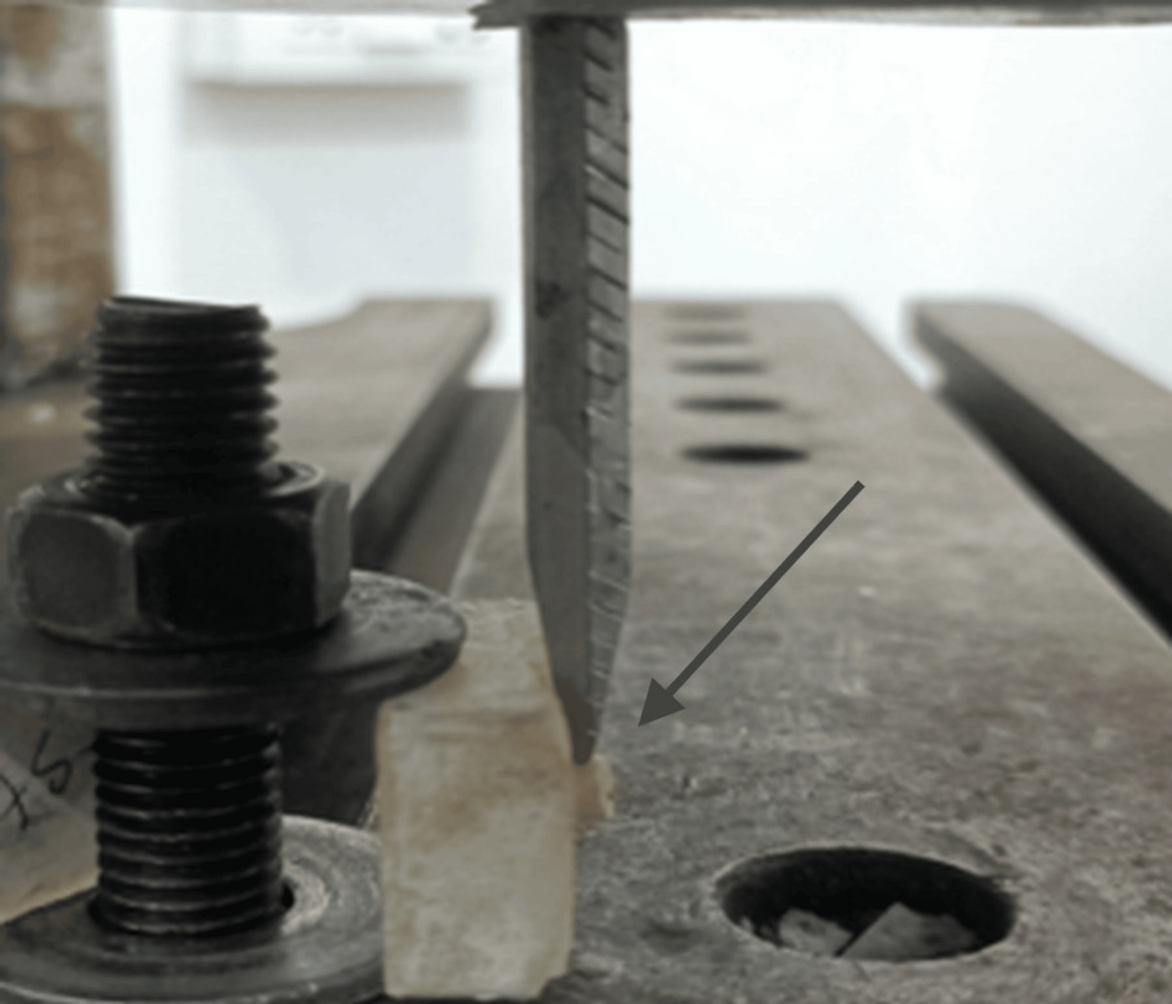
Measurement of shear bond strength (SBS) using a universal testing machine. The arrow indicates the shear blade applying load at the enamel-sealant interface.

After sealant application, all specimens were stored in distilled water at 37°C for 24 hours and thermocycled (1,000 cycles between 5°C and 55°C, 30-second dwell time). SBS was measured using a universal testing machine at a crosshead speed of 1 mm/min (Figure 2).

Statistical analysis

All collected data were entered and analyzed using IBM SPSS Statistics for Windows, Version 26.0 (IBM Corp., Armonk, NY, USA). Descriptive statistics (mean, SD, minimum, and maximum) were calculated for each group. Intergroup comparisons of mean SBS values were performed using one-way ANOVA. When statistically significant differences were found, Tukey’s Honest Significant Difference (HSD) post hoc test was employed for pairwise comparisons. The level of significance was set at p < 0.05.

## Results

The results presented in Table 1 suggest that Group 3 (Er:YAG laser pre-conditioning, phosphoric acid etching, followed by UltraSeal XT® hydro™) exhibits the highest mean shear bond strength compared to all other tested groups. The relatively low variability (SD) and narrow range (min-max) within each group indicate good consistency of the measurements. Further statistical analysis, including ANOVA (Table 2), was applied to determine whether the observed differences in mean shear bond strength were statistically significant.

**Table 1:** Descriptive statistics comparing mean shear bond strength among groups.

Group	N	Mean (MPa)	SD	Min	Max
Group 1	10	20.4	0.99	18.6	22.2
Group 2	10	19.6	0.98	17	20.7
Group 3	10	22.3	0.98	19.5	23.1
Group 4	10	20.8	0.98	18	21.6

**Table 2:** One-way ANOVA for comparison of mean shear bond strength among groups. *** denotes highly significant (p < 0.001).

Source	Sum of Squares	df	Mean Square	F	p-value
Between Groups	31.8	3	10.6	11	< 0.001***
Within Groups	34.85	36	0.97	-	-

From the ANOVA table, since the p-value is less than 0.05 (the level of significance), we reject the null hypothesis and conclude that there is a significant difference in mean shear bond strength among the groups. Furthermore, to assess specific pairwise comparisons, Tukey’s post hoc test (Table 3) was applied.

**Table 3:** Post hoc test for pairwise comparison (Tukey’s test).

Group-wise Comparison	Mean Difference	t	Tukey Test (p-value)	Decision
Group 1 - Group 2	0.816	1.855	0.0718	Insignificant
Group 1 - Group 3	-1.938	-4.405	< 0.001	Significant
Group 1 - Group 4	-0.418	-0.95	0.348	Insignificant
Group 2 - Group 3	-2.705	-6.148	< 0.001	Significant
Group 2 - Group 4	-1.203	-2.734	0.009	Significant

The pairwise comparison of shear bond strength across the groups indicated significant variations among the tested groups. Group 1 demonstrated a higher average shear bond strength compared to Group 2, although the difference was not statistically significant (mean difference = 0.816, p = 0.0718). Group 3 exhibited statistically significant and higher shear bond strength than Group 1 (mean difference = -1.938, p < 0.001), Group 2 (mean difference = -2.705, p < 0.001), and Group 4 (mean difference = 1.516, p = 0.001). Group 1 and Group 4 showed similar bond strengths (mean difference = -0.418, p = 0.348), with no statistically significant difference between them. However, Group 4 demonstrated statistically significant and higher shear bond strength than Group 2 (mean difference = -1.203, p = 0.009).

## Discussion

The current study evaluated the effect of Er:YAG laser pre-conditioning on the SBS of a hydrophilic fissure sealant (UltraSeal XT® hydro™) under both dry and moisture-contaminated conditions. The results demonstrate that Er:YAG laser pre-conditioning, in combination with conventional acid etching, significantly improves SBS, particularly in scenarios where moisture contamination is present.

UltraSeal XT® hydro™ is designed to tolerate slight moisture; yet, our findings align with previous studies suggesting that even hydrophilic sealants are not entirely immune to the detrimental effects of saliva contamination. Panigrahi A et al. reported that hydrophilic sealants exhibited reduced bond strength when applied to saliva-contaminated enamel, corroborating the observed decrease in SBS for Group 2 (acid etching + saliva contamination) in our study [6]. Similarly, Shimazu K and Bao Z et al. highlighted that contamination alters enamel surface energy and reduces resin infiltration, thereby undermining sealant retention [3,4].

Group 3 (Er:YAG + acid etching, dry conditions) exhibited the highest SBS, indicating that laser pre-treatment substantially enhances enamel bonding. This improvement is likely due to laser-induced micro-roughening, the formation of prism-free surfaces, and the removal of the enamel smear layer, which collectively enhance micromechanical retention and resin penetration [7-10]. Revdekar AH et al. confirmed that Er:YAG laser irradiation produces uniform micro-retentive enamel morphology, facilitating stronger adhesive interfaces [10]. Sungurtekin-Ekçi E et al. and Rattanacharoenthum A et al. also reported that laser-treated enamel increases surface energy and wettability, thereby improving resin adaptation [8,9].

Interestingly, Group 4 (Er:YAG + acid etching + saliva contamination) demonstrated significantly higher SBS than Group 2 (acid etching + saliva contamination), although lower than Group 3. This suggests that laser pre-conditioning can partially mitigate the adverse effects of moisture contamination. Yilmaz H and Keles S observed similar findings in clinical trials, reporting improved sealant retention with Er:YAG pre-conditioning even when absolute moisture control was not achievable [12]. Nevertheless, complete compensation for contamination was not observed, emphasizing the persistent challenge that saliva presents to adhesion.

Group 1 (acid etching, dry) and Group 4 (laser + acid etching + saliva contamination) showed intermediate SBS values, highlighting that while conventional acid etching provides adequate adhesion under ideal conditions, laser-assisted pre-conditioning enhances bond strength and offers resilience against moisture. Zhang Y and Jiang A's systematic review and meta-analysis further support this observation, concluding that Er:YAG laser pre-conditioning consistently improves bond strength across enamel substrates compared with acid etching alone [11].

Clinical implications

From a clinical perspective, these results underscore the potential of laser-assisted enamel preparation as a supplementary strategy to improve sealant retention, particularly in pediatric patients or uncooperative individuals where moisture control is challenging. While hydrophilic sealants such as UltraSeal XT® hydro™ provide some tolerance to contamination, their performance can be significantly enhanced by Er:YAG laser pre-treatment. Additionally, the combination of laser and acid etching appears to optimize the enamel surface for both mechanical interlocking and chemical bonding, thereby improving long-term retention and potentially reducing the risk of secondary caries [5].

Limitations and future directions

This study is limited by its laboratory-based design, which may not fully replicate the oral environment with its dynamic pH, temperature fluctuations, and masticatory forces. The use of artificial saliva may not precisely mimic natural saliva in terms of viscosity and enzymatic composition. Furthermore, variability in tooth storage conditions and extraction-to-testing intervals could have influenced enamel properties. Future investigations should include in vivo clinical trials, assess long-term microleakage, and explore optimal laser parameters for various enamel conditions. The development of next-generation hydrophilic sealants specifically tailored for laser-conditioned enamel may further improve clinical outcomes.

## Conclusions

Within the limitations of this in vitro study, Er:YAG laser pre-conditioning significantly enhanced the SBS of a hydrophilic fissure sealant, even under conditions of moisture contamination. This approach may provide a clinically relevant method to improve sealant retention in cases where ideal moisture control is difficult to achieve. However, as these results are derived from controlled laboratory conditions, further split-mouth randomized clinical trials are strongly recommended to validate these findings in vivo and to evaluate long-term clinical outcomes such as sealant retention, microleakage, and caries prevention.
